# Estrogen Receptor-α36 Mediates EGFR-SGK1 Signaling-Related Erk Activation in Gastric Cancer

**DOI:** 10.3390/cells15090787

**Published:** 2026-04-26

**Authors:** Yibo Zhang, Hongyan Zhou, Yifan Xiao, Shubing Yang, Qingqing Xu, Xin Liu, Wenli Huang, Mingshan Pi, Qi Xiong, Xiaochuan Wang, Xiji Shu, Yiyuan Xia

**Affiliations:** 1 Hubei Key Laboratory of Cognitive and Affective Disorders, School of Medicine, Jianghan University, No. 8 Sanjiaohu Road, Wuhan 430056, China; 17613058002@163.com (Y.Z.); zhouhongyan517@163.com (H.Z.); yfxiao@jhun.edu.cn (Y.X.); yangshubing2023@163.com (S.Y.); xqq870@outlook.com (Q.X.); liuxin991026@163.com (X.L.); 17862890905@163.com (W.H.); 13419560822@163.com (M.P.); xiongq@jhun.edu.cn (Q.X.); wxch@tjmu.edu.cn (X.W.); xijishu@jhun.edu.cn (X.S.); 2 Institutes of Biomedical Sciences, School of Medicine, Jianghan University, No. 8 Sanjiaohu Road, Wuhan 430056, China; 3 Department of Pathology and Pathophysiology, School of Medicine, Jianghan University, No. 8 Sanjiaohu Road, Wuhan 430056, China

**Keywords:** gastric cancer, ER-α36, epidermal growth factor receptor, serum and glucocorticoid-regulated kinase 1, MAPK signaling pathway

## Abstract

Introduction: Gastric cancer is a prevalent and aggressive malignancy driven by complex signaling networks. Estrogen receptor-α36 (ER-α36), a membrane-localized receptor, mediates non-genomic signaling and promotes tumor progression. ER-α36 can interact with epidermal growth factor receptor (EGFR) to activate downstream mitogen-activated protein kinase (MAPK) signaling, but the detailed mechanism in gastric cancer remains unclear. This study aimed to explore whether ER-α36 promotes gastric cancer progression by regulating serum and glucocorticoid-regulated kinase 1 (SGK1)-mediated Erk1/2 activation.Methods:We collected 53 human gastric adenocarcinoma specimens and detected ER-α36 expression by immunohistochemistry. Bioinformatics analysis was used to identify ER-α36-related kinases. Gastric cancer cell lines (SGC7901, HGC27, NCI-N87, and MFC) were used for in vitro studies. Western blotting, qRT-PCR, immunofluorescence, co-immunoprecipitation (Co-IP), wound healing, MTT, and Transwell invasion analyses, and nude mouse orthotopic tumor models were applied to investigate the function and mechanism of the ER-α36/SGK1/Erk1/2 axis. Results: ER-α36 was positively expressed in 62.3% of gastric adenocarcinoma tissues and was associated with poor differentiation and prognosis. SGK1 was identified as a key kinase downstream of ER-α36. ER-α36, SGK1, and p-Erk1/2 were co-upregulated in gastric cancer tissues and cells. ER-α36 regulated Raf/MEK1/2/Erk1/2 phosphorylation in an SGK1-dependent manner. EGF-induced Erk1/2 activation required both ER-α36 and SGK1. Overexpression of ER-α36 promoted the proliferation, migration, and invasion of gastric cancer cells, while SGK1 knockdown abolished these oncogenic effects. In vivo experiments confirmed that ER-α36 promoted gastric tumor growth and EGFR/Erk signaling, which was attenuated by SGK1 knockdown. Conclusions: ER-α36 contributes to the malignant progression of gastric adenocarcinoma by activating the Erk1/2 pathway through SGK1. The ER-α36–SGK1–Erk1/2 axis may serve as a novel therapeutic target for gastric cancer.

## 1. Introduction

Gastric cancer is a prevalent and aggressive malignancy of the digestive tract, with a complex pathogenesis involving multiple signaling pathways. Estrogen receptors (ERs) have been recognized as important factors in gastric cancer development. Notably, a growing number of clinical studies, including a recent case series from a Western center, have reported the expression of estrogen receptor alpha (ERα) in a subset of gastric cancers and demonstrated its association with patient prognosis and histopathological characteristics, suggesting that ERα may contribute to the malignant behaviors of gastric cancer cells [1]. Among the ERα subtypes, ER-α36 has recently emerged as a focal point in cancer research. Distinct from the classical nuclear ERα, ER-α36 is predominantly membrane-localized, which enables rapid non-genomic signaling. It has been demonstrated that ER-α36 can interact with the epidermal growth factor receptor (EGFR), and this interplay may be pivotal in driving the growth and metastasis of malignant tumors [2]. Moreover, the high expression of ER-α36 in certain ER-negative tumors underscores its promise as a new therapeutic target for refractory cancers [3].

The epidermal growth factor receptor (EGFR) functions as a core regulator governing cell proliferation, differentiation, and survival. Dysregulation and aberrant activation of EGFR are widely implicated in the initiation and progression of multiple human malignancies. EGFR forms homo- or heterodimers with other ErbB family members (HER2, ErbB3, and ErbB4) upon ligand stimulation, triggering autophosphorylation and preferential activation of downstream signaling pathways including MAPK, PI3K, and STAT3. High-affinity ligands such as EGF induce stable receptor dimers, whereas low-affinity ligands (EREG and EPGN) give rise to unstable dimers but promote more sustained phosphorylation at specific tyrosine residues. Distinct ligand-dependent EGFR activation patterns further lead to differential downstream signaling outcomes. Parallel investigations in papillary thyroid carcinoma (PTC) and non-invasive follicular thyroid neoplasms have extensively characterized EGFR expression dynamics and its regulatory relationships with key microRNAs, highlighting its pivotal role in thyroid tumorigenesis [4].

Recent studies have shed light on the ability of ER-α36 to activate downstream signaling cascades, such as the mitogen-activated protein kinase (MAPK) pathway [5], through its interaction with EGFR. This activation cascade directly promotes oncogenic phenotypes, enhancing both proliferative capacity and migratory potential of malignant cells [6]. In the context of gastric cancer, Erk activation has been closely linked to tumor aggressiveness and patient prognosis, underscoring the clinical relevance of exploring the relationship between ER-α36 and EGFR. Such investigations may not only enhance our understanding of tumor biology, but also pave the way for innovative therapeutic approaches [7].

In parallel, the glucocorticoid-inducible kinase SGK1, which participates in growth promotion and cell survival mechanisms, has garnered increasing attention. Emerging evidence suggests that SGK1 is a key player in tumor initiation and progression. It has been identified as a significant regulator in fibrotic diseases, orchestrating tissue remodeling and cellular signaling pathways [8]. Moreover, SGK1 exhibits aberrant expression patterns in various cancers, with its overexpression being closely associated with enhanced tumor cell survival, migration, and invasiveness. This has positioned SGK1 as a promising therapeutic target, particularly in aggressive malignancies such as pancreatic and breast cancers [9,10]. Additionally, SGK1 has been shown to play a crucial role in regulating cellular metabolism and apoptosis resistance, further solidifying its potential as a therapeutic target [11,12].

SGK1 modulates the activity of epidermal growth factor receptor (EGFR)-related signaling pathways through multiple mechanisms, thereby exerting profound effects on a variety of cellular processes, including proliferation, migration, and survival [13]. Importantly, this regulatory function is not exerted in isolation but is closely integrated with core cellular events such as autophagy, thereby coordinately contributing to the establishment and progression of malignant phenotypes. A growing body of evidence indicates that the functional crosstalk between SGK1 and EGFR is widely involved in the development of various malignant tumors. In breast cancer, phylogenetic analysis has identified concurrent mutations in SGK1 and EGFR, suggesting a potential functional interaction between these two kinases during tumorigenesis and malignant progression [14]. In lung cancer, driver mutations such as EGFR exon 20 insertions serve as key events driving malignant transformation, while aberrantly activated SGK1 acts as a critical downstream effector that promotes tumor invasion and metastasis [15]. Collectively, these studies support the conclusion that SGK1 functions as a tumor-promoting regulator in multiple cancer types by modulating EGFR signaling activity [16,17]. This oncogenic mechanism has also been validated in gastric cancer cells. SGK1 has been demonstrated to enhance the growth and survival of gastric cancer cells by potentiating EGFR signaling. Notably, SGK1 ablation significantly inhibits the proliferation and migration of gastric cancer cells, an effect that is tightly correlated with reduced EGFR pathway activation, further highlighting the essential role of SGK1 in gastric cancer progression [18].

The present study delves into whether ER-α36 activates the downstream Erk pathway by upregulating SGK1. Through database mining of ER-α36-related protein networks, SGK1 was identified as the sole kinase. A positive correlation between SGK1 expression and pathological grading of tumor samples was observed, and it was validated that SGK1 can directly phosphorylate Erk1/2, thereby enhancing gastric cancer cell activity. By dissecting the signaling interplay among ER-α36, EGFR, and SGK1, this study offers a novel perspective on gastric cancer research.

## 2. Methods

### 2.1. Case Selection

Specimens were obtained from 53 patients who underwent curative resection of gastric cancer at the pathology institution of Jianghan University, China, between 1 July 2007 and 1 November 2009. The patients comprised 45 males and 8 females, aged 36–79 years (mean 57.8). Tumor size, histological grade, T classification, and N classification were assessed based on the 2003 WHO clinicopathological criteria. This experiment was approved by the Medical Ethical Committee of Jiang Han University (approval JHDXKJLL2025-131).

### 2.2. Materials and Reagents

Anti-Erk1/2 antibody was purchased from TDY-bio (Beijing, China; TDY491). Anti-phospho-Erk1/2 (Thr202/Tyr204) antibody was obtained from Cell Signaling Technology (4370). Antibodies against SGK1 (YM9012), EGFR (YM8344), Raf-1 (YM8477), phospho-Raf1 (Ser259, YM9067), MEK1/2 (YM8273), and phospho-MEK1/2 (Ser217/221, YM8556) were purchased from Immunoway (San Jose, CA, USA). Recombinant human EGF was obtained from MCE (HY-P7109, Monmouth Junction, NJ, USA). Rabbit anti-ER-α36 antibody was kindly provided by Dr. Zhao-Yi Wang, Department of Medical Microbiology, Immunology, and Pathology, Creighton University Medical School, USA.

### 2.3. Cell Culture

The human gastric cancer cell lines SGC7901, HGC-27, and NCI-N87 were used in this study. SGC7901 cells were provided by the Department of Immunology, Tongji Medical College (Wuhan, China). HGC-27 (C6365) and NCI-N87 (C6705) cells were purchased from Beyotime Biotechnology (Shanghai, China). SGC7901 and HGC-27 cells were cultured in DMEM supplemented with 10% fetal bovine serum (FBS). NCI-N87 cells were maintained in RPMI-1640 medium containing 10% FBS. All cells were cultured at 37 °C in a humidified atmosphere with 5% CO_2_.

Stable ER-α36-overexpressing SGC7901 and HGC27 cell lines were established using lentivirus rLV-CMV-Humaner-ER-α36-HA-Luciferase-Puro. SGK1 knockdown was performed in ER-α36-overexpressing SGC7901 cells using rLV-U6-shRNA (human SGK1)-Bla For ER-α36 knockdown, small interfering RNA (siRNA) targeting ER-α36 was transfected into SGC7901 cells using a lipofectamine-based transfection reagent. Cells were collected for subsequent assays 48 h after transfection. All lentiviruses were constructed by Design Gene Biotechnology (Shanghai, China).

MFC gastric cancer cells (Jianghan University Medical Department) were cultured in RPMI-1640/10% FBS at 37 °C with 5% CO_2_. We used the plasmid pcDNA3.1-ER-α36-3×Flag to transfect and overexpress ER-α36, and similarly, we used small interfering RNA (siRNA) transfection to knock down ER-α36 expression. Likewise, we used pcMV-SGK1-3Flag-Neo transfection to overexpress SGK1 levels and siRNA transfection to knock down SGK1 expression. After 48 h of siRNA-SGK1 treatment, EGF (10 nM) stimulation was administered for 10 min before total protein extraction from the cells [19].

### 2.4. Immunoprecipitation

Cells were cultured to 70–90% confluence, washed twice with pre-chilled PBS, and lysed with pre-chilled 1% NP-40 lysis buffer on ice for 10 min, followed by further lysis on a rotator at 4 °C for 30 min. The lysates were centrifuged at 12,000 rpm for 15 min at 4 °C, and the supernatant (total protein lysate) was collected. The total protein lysate was divided into input, IgG control, and SGK1 IP groups. Normal IgG and anti-SGK1 primary antibody were added to the IgG and IP groups, respectively, followed by overnight incubation on a rotator at 4 °C. Protein A/G magnetic beads were pre-washed four times with PBST (5–10 min each) on a magnetic stand, and then added to the IgG and IP groups for binding. The bead complexes were washed with PBST (10 min each), and then 50 μL of 1× Loading buffer (diluted with IP lysis buffer) was added. Samples were boiled at 100 °C for 5 min, the magnetic beads were removed, and the supernatant was retained for subsequent analysis.

### 2.5. Wound Healing Assay

Cells were seeded into 6-well plates and cultured to full confluence. Adding 2 ug/mL Mitomycin C (TargetMol, T6890, Boston, MA, USA) at 37 °C for 1 h reduced cell proliferation. A linear scratch was created in the confluent monolayer using a sterile 200 μL pipette tip. After washing with PBS to remove floating cells, serum-free medium was added. Wound closure was photographed at 0, 24, and 48 h using an inverted microscope. Migration distance was measured using ImageJ software(Version 1.54f, National Institutes of Health, Bethesda, Maryland, MD, USA), and relative mobility was calculated.

### 2.6. MTT Assay

Stably transfected cells were seeded into 96-well plates at a density of 2000 cells per well. Then, 10 μL of MTT reagent was added to each well, and cells were incubated at 37 °C for 4 h. Subsequently, 100–150 μL of DMSO was added to dissolve the formazan crystals. The absorbance at 570 nm was measured using a microplate reader. Cell viability was expressed as a percentage relative to the control group.

### 2.7. Transwell Migration Assay

Cells suspended in serum-free medium were added to the upper chamber of Transwell plates. The lower chamber was filled with complete medium containing 10% FBS. After 24 h of incubation, cells were fixed with 4% paraformaldehyde at 4 °C for 30 min and stained with 0.1% crystal violet for 30 min. Non-invading cells on the upper surface were removed with cotton swabs. Five random fields per chamber were photographed and counted.

### 2.8. Nude Mouse Orthotopic Transplantation Model

Male BALB/c nude mice (4 weeks old) were purchased from Beijing HFK Bioscience Co., Ltd. Mice were randomly divided into groups. Under isoflurane anesthesia, 1 × 10^6^ cells suspended in 20 μL PBS were injected into the gastric wall of nude mice using a 29G needle. Tumor growth was monitored by small animal in vivo bioluminescence imaging. Mice were sacrificed on day 28, and tumors were dissected for further analysis.

### 2.9. Immunofluorescence

Using a microtome, paraffin-embedded human gastric adenocarcinoma specimens obtained from the Pathology Research Institute of Jianghan University were sectioned into 4 μm thick sections. After floating the sections in a water bath, they were mounted onto adhesive glass slides and dried on a slide warmer to ensure adhesion. Following deparaffinization in xylene, the sections were rehydrated via a graded alcohol series. The tissue sections underwent antigen retrieval using an antigen retrieval solution (sodium citrate dihydrate (2 g) and citric acid monohydrate (0.3 g) dissolved in distilled water to a 1 L final volume) in a 60 °C water bath for 30 min, followed by natural cooling. The sections were washed with PBS for 5 min, which was repeated three times. Endogenous peroxidase activity was blocked by adding a blocking reagent and incubating under dark conditions at 22–25 °C for 25 min. Sections were incubated with a 3% BSA blocking solution (30 min at 22 °C) to minimize background staining. Sections were incubated with primary antibodies overnight at 4 °C under light-protected conditions. After incubation, the sections were washed three times with PBS, and tissues were incubated with species-matched HRP-conjugated secondary antibodies, followed by incubation in the dark at room temperature for 50 min. TYR dyes (TYR-480, TYR-520, TYR-570) were added for 2–15 min, and the sections were washed three times with PBS. The antigen retrieval process was repeated, and all steps were performed again twice. Finally, an anti-fade mounting medium containing DAPI was applied, and the sections were covered with a coverslip for microscopic examination.

### 2.10. Immunohistochemical Staining

Slices with a thickness of 4 microns were dewaxed in xylene, and antigens were retrieved using EDTA microwave heating. Endogenous peroxidase was blocked with hydrogen peroxide, followed by blocking with 5% BSA for 30 min. The sections were incubated with primary antibodies at room temperature for 2 h and then with secondary antibodies for 30 min. Staining was visualized using DAB chromogen. Hematoxylin was used for counterstaining. Sections were dehydrated through a graded alcohol series and mounted. For IHC analysis, the staining intensity was given a grade of 0–3 and the proportion of positive cells was given a grade of 0–4. An H-score (intensity × proportion) ranging from 0 to 12 was used for quantification. Cases with an H-score above the median were defined as high expression, and those below it as low expression. Samples with no staining (H-score = 0) were considered negative.

### 2.11. Quantitative Real-Time PCR Assay

Total RNA was extracted from tissues or cells using a commercial RNA extraction kit (R30506, Shanghai Yeyuan Biotechnology Co., Ltd., Shanghai, China). One microgram of RNA was reverse-transcribed into cDNA using the PrimeScript II 1st Strand cDNA Synthesis Kit (Takara, 6210A, Kusatsu, Japan). qRT-PCR was performed using TB Green Fast qPCR Mix (Takara, RR420) on a CFX96 real-time PCR system (Applied Biosystems, Foster City, CA, USA). Relative gene expression was calculated using the 2^−ΔΔCt^ method.

The primer sequences were as follows:

β-actin: Forward 5′-AGATCCCTCCAAAATCAAGTGG-3′;

Reverse 5′-GGCAGAGATGATGACCCTTTT-3′;

ERα-36: Forward 5′-AGGAGGAAGAGCTGCCAGGC-3′;

Reverse 5′-GAAAAGACCGAAGAGGAGGG-3′;

SGK1: Forward 5′-AGGGCAGTTTTGGAAAGGTT-3′;

Reverse 5′-CTGTAAAACTTTGACTGCATAGAACA-3′;

MAPK1: Forward 5′-ACTCCTTTGAGCCGTTTGGA-3′;

Reverse 5′-GGTCAGCAGGGCATCATGTA-3′.

### 2.12. Western Blotting Detection

Transfected cells were harvested and lysed in RIPA buffer on ice for 20 min. After sonication to disrupt cell membranes, lysates were centrifuged at 4 °C, and the supernatant was collected. Protein concentration was determined using a protein quantification kit (Abbkine, Wuhan, China, KTD3001). Following SDS-PAGE electrophoresis and membrane transfer, nonspecific binding sites were blocked with 5% non-fat milk at room temperature for 1 h. Membranes were washed twice with TBST (5 min each wash). Primary antibodies were diluted according to the manufacturer’s instructions and incubated with the membranes at 4 °C overnight with gentle shaking. The next day, the membranes were washed three times with TBST to remove unbound primary antibodies, incubated with species-matched horseradish peroxidase (HRP)-conjugated secondary antibodies at room temperature for 1 h, and then washed three additional times with TBST. Protein bands were visualized using ECL chemiluminescent substrate and imaged with a chemiluminescence imaging system.

### 2.13. Bioinformatics Analysis

Volcano plot data were obtained from the GSA database (accession No. HRA001689). Kaplan–Meier survival curves for ER-α36 (encoded by ESR1) and SGK1 in gastric adenocarcinoma were generated using the GEPIA2 database. KEGG pathway enrichment analysis of the differentially expressed genes (DEGs) associated with ESR1 and SGK1 was performed using the TCGA database.

### 2.14. Statistical Analysis

All experiments were performed with at least three independent biological replicates and three technical replicates per sample to ensure reproducibility. Quantitative data are presented as mean ± standard deviation (SD) and visualized using GraphPad Prism (version 8.0) software.

Fluorescence intensity quantification: The fluorescence intensity of ER-α36, SGK1, and p-Erk1/2 was analyzed using ImageJ software with the Integrated Density and Colocalization Analysis plugins. For cellular immunofluorescence, the relative fluorescence intensity of target proteins was normalized to the fluorescence intensity of DAPI in the same field to eliminate variations caused by differences in cell number or tissue thickness. For tissue sections, the average fluorescence intensity of gastric cancer tissues was normalized to that of adjacent non-tumorous tissues in the same section, which served as an internal control.

Western blot quantification: Densitometric analysis of protein bands was performed using ImageJ software. The relative expression level of each target protein was normalized to the loading control (β-actin) and calculated as the ratio of the target protein band intensity to the β-actin band intensity in the same sample.

In vitro and in vivo functional assays: For time-dependent effects (MTT cell viability assay over 24/48/72 h), statistical significance was determined using two-way analysis of variance (ANOVA) followed by Bonferroni post hoc testing. For comparisons between two groups (control vs. ER-α36-overexpressing cells, control vs. SGK1-knockdown cells), the unpaired two-tailed Student’s *t*-test was used. For comparisons among three or more groups, one-way ANOVA followed by Tukey’s post hoc test was applied. A two-sided *p* value < 0.05 was considered statistically significant.

## 3. Results

### 3.1. SGK1 Is a Differentially Expressed Gene Mediating Multiple Signaling Pathways in Gastric Adenocarcinoma

**Figure 1:**
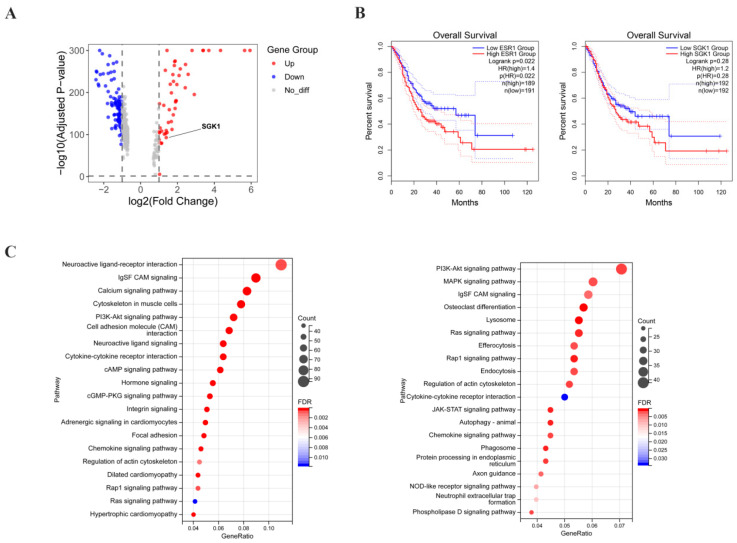
The expression level of SGK1 changes in gastric adenocarcinoma, and it mediates many signal pathways. (**A**) Volcano map of differentially expressed genes. Single-cell RNA sequencing of gastric cancer tissues showed that 48 genes, including SGK1, were upregulated (red) and 112 genes, including CLDN3, were downregulated (blue) in gastric cancer cells relative to normal gastric mucosal cells; gray represents non-differentially expressed genes. The *x*-axis represents the log fold change (log2FC) in expression, and the *y*-axis represents statistical significance (−log_10_ *p* value). The SGK1 gene is highlighted. (**B**) The Kaplan–Meier survival curves for ESR1 and SGK1 were plotted using the log-rank test in GEPIA2. (**C**) Transcriptomic data from 410 TCGA-STAD gastric cancer samples were stratified into high- and low-expression groups based on the median TPM of ESR1 and SGK1. Differential expression analysis showed that upregulated genes in the ESR1 high-expression group were significantly enriched in the PI3K-AKT and Ras pathways, whereas those in the SGK1 high-expression group were enriched in the PI3K-AKT, MAPK, and Ras pathways. These findings suggest that ESR1 and SGK1 may cooperatively regulate the MAPK signaling pathway in gastric cancer.

To characterize ER-α36 expression in gastric adenocarcinoma specimens, immunohistochemistry (IHC) was performed on specimens from 53 gastric cancer patients. ER-α36 was detected in 33 of 53 (62.3%) primary gastric adenocarcinoma samples. Most importantly, statistical analysis identified a significant association between ER-α36 expression and histological differentiation (*p* = 0.02). Specifically, the positive rate and expression level of ER-α36 were markedly increased in poorly differentiated tumors compared with well-differentiated cases, indicating that ER-α36 positivity significantly increases with decreasing histological differentiation. In contrast, there were no significant associations between ER-α36 expression and other parameters, including gender, age, tumor size, T stage, or lymph node metastasis (Table 1). Previous studies have shown that the expression level of SGK1 was significantly altered in gastric adenocarcinoma tissues, and this gene mediated the regulation of multiple signaling pathways. To screen for key genes involved in gastric adenocarcinoma progression, we first analyzed genome-wide expression profiles. A volcano plot was constructed to visualize differentially expressed genes (DEGs), which demonstrated that SGK1 was a significantly differentially expressed gene in gastric adenocarcinoma (Figure 1A). SGK1 was thus identified as a candidate gene for further investigation. Survival analysis was performed to assess the prognostic value of ESR1 and SGK1 in gastric cancer. High expression of ESR1 tended to be associated with poor overall survival. Similarly, the cumulative survival rate of the SGK1 high-expression group was slightly lower than that of the low-expression group, although the separation between the two curves was not obvious. The survival curves of the SGK1 high- and low-expression groups showed no statistically significant difference in overall survival, but a trend toward worse survival was observed in the high-expression group (Figure 1B). ESR1 encodes multiple ERα isoforms, including ERα66 and ER-α36. Since TCGA mRNA data do not distinguish individual isoforms, no conclusion can be made regarding ER-α36 protein level and patient survival. High ER-α36 expression is associated with poor prognosis. However, combined with the observation from Table 1 that high ER-α36 expression is associated with poor histological differentiation, we speculate that ER-α36 may drive the malignant phenotype of gastric cancer partly by regulating downstream molecules including SGK1. KEGG pathway enrichment analysis was performed using TCGA-STAD transcriptomic data to identify signaling pathways associated with ESR1 and SGK1. Differentially expressed genes (DEGs) in the ESR1 high-expression group and SGK1 high-expression group were both significantly enriched in several key oncogenic pathways, including neuroactive ligand–receptor interaction, and the PI3K-Akt, MAPK, and Ras pathways. In the bubble plot, the enrichment significance was ranked by the false discovery rate (FDR), the size of each bubble represents the number of enriched DEGs, and the color intensity corresponds to the enrichment significance (−log_10_ FDR) (Figure 1C). These results suggest that ESR1 and SGK1 may jointly regulate the MAPK/Erk signaling cascade during gastric cancer progression, which further supports the functional link between ESR1, its isoform ER-α36 (as validated in Table 1), and SGK1 in gastric cancer pathogenesis (Figure 1C).

### 3.2. Co-Upregulation of ER-α36, SGK1, and p-Erk1/2 in Gastric Adenocarcinoma and Their Correlation with Histological Differentiation

**Figure 2:**
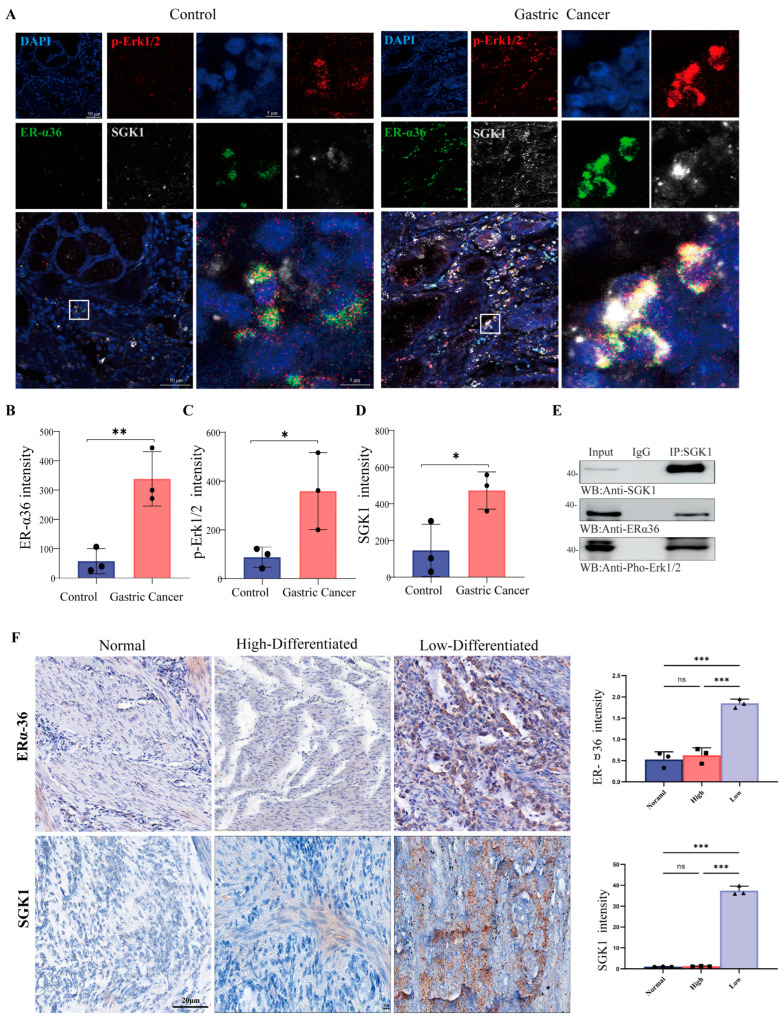
ER-α36, SGK1 and p-Erk1/2 are simultaneously upregulated in human gastric adenocarcinoma tissues and cell lines, and correlate with histological differentiation. (**A**) Representative immunofluorescence staining showing the subcellular localization and co-expression of ER-α36 (green), SGK1 (gray), and p-Erk1/2 (red) in adjacent non-tumor gastric mucosal tissues (Control) and gastric adenocarcinoma tissues (Cancer). Nuclei were counterstained with DAPI (blue). The white square marks the magnified region of interest (ROI) shown in the corresponding panel. Scale bar: 50 μm. (**B**–**D**) Quantification of ER-α36 (**B**), p-Erk1/2 (**C**), and SGK1 (**D**) fluorescence intensity in patient tissues. Fluorescence signals were quantified using ImageJ software and normalized to paired peritumoral controls. Statistical analysis was performed using an unpaired two-tailed Student’s *t*-test. (**E**) Co-immunoprecipitation (Co-IP) assay verifying the physical interaction between ER-α36, p-Erk and SGK1 in gastric cancer cells. Whole-cell lysates (Input) were immunoprecipitated with an anti-SGK1 antibody, followed by immunoblotting with antibodies against ER-α36 and SGK1. Normal IgG was used as a negative control for immunoprecipitation. (**F**) Representative immunohistochemical (IHC) staining of ER-α36 and SGK1 in normal, high-differentiated and low-differentiated gastric adenocarcinoma tissues. Scale bar: 20 μm. Expression levels were quantified by relative intensity and presented as histograms. Data were normalized to peritumoral controls and analyzed using an unpaired two-tailed Student’s *t*-test. All quantitative data are presented as the mean ± SD from three independent biological replicates. * *p* < 0.05, ** *p* < 0.01, *** *p* < 0.001.

To investigate the clinical expression patterns and potential functional association of ER-α36, SGK1, and the MAPK/ERK pathway in human gastric cancer, we first examined their expression levels in clinical gastric adenocarcinoma specimens. We observed that ER-α36, SGK1, and p-Erk1/2 were coordinately upregulated in gastric adenocarcinoma tissues compared with adjacent non-tumorous control tissues. Immunofluorescence staining further revealed distinct subcellular localization and co-expression patterns of ER-α36, SGK1, and p-Erk1/2 in cancer tissues (Figure 2A). Quantitative fluorescence intensity analysis confirmed that the expression levels of all three proteins were significantly elevated in tumor tissues relative to peritumoral controls (Figure 2B–D; * *p* < 0.05, ** *p* < 0.01, *** *p* < 0.001). Given their coordinated upregulation, we next examined whether ER-α36, p-Erk and SGK1 physically interact in gastric cancer cells. Co-immunoprecipitation (Co-IP) assays verified a direct protein–protein interaction between ER-α36 and SGK1 (Figure 2E).

We further explored the clinical significance of ER-α36 and SGK1 by analyzing their expression in relation to histological differentiation status. The expression levels of ER-α36 and SGK1 are closely related to the histological differentiation of gastric adenocarcinoma. Immunohistochemical (IHC) staining showed that the expression levels of ER-α36 and SGK1 in poorly differentiated gastric adenocarcinoma were higher than those in well-differentiated gastric adenocarcinoma. Quantitative analysis confirmed that their expression levels in gastric adenocarcinoma tissues with different degrees of differentiation were statistically significant (Figure 2F) (* *p* < 0.05, ** *p* < 0.01, *** *p* < 0.001).

### 3.3. ERα-36 Modulates Raf/MEK1/2/Erk1/2 Phosphorylation via SGK1 in Gastric Cancer Cells

To identify appropriate cellular models for mechanistic investigation, we first evaluated the basal expression levels of EGFR, ER-α36, and SGK1 across three human gastric cancer cell lines. Western blot analysis demonstrated that HGC27, SGC7901, and NCI-N87 cells exhibited heterogeneous basal expression of these three proteins; β-actin was used as the loading control (Figure S1; * *p* < 0.05, ** *p* < 0.01, *** *p* < 0.001). This observed heterogeneity justified the use of multiple cell lines to ensure the robustness and reproducibility of subsequent mechanistic studies.

**Figure 3:**
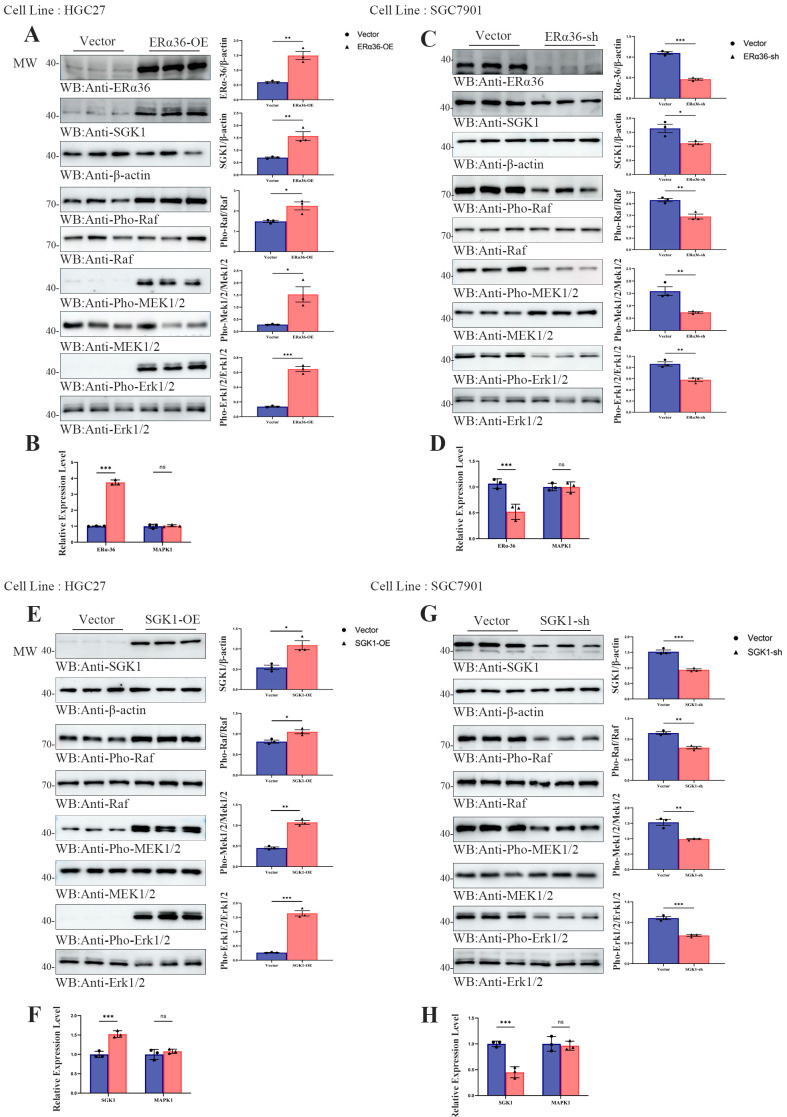
ER-α36 promotes Erk1/2 phosphorylation via SGK1 in gastric cancer cells. (**A**) Western blot analysis showing the overexpression efficiency of ER-α36 and its effect on SGK1 expression, as well as the phosphorylation of Raf, MEK1/2, and Erk1/2 in HGC27 cells. β-actin was used as the loading control. (**B**) qRT-PCR analysis of the overexpression efficiency of ER-α36 and the mRNA levels of MAPK1 following ER-α36 overexpression in HGC27 cells. (**C**) Western blot analysis showing the knockdown efficiency of ER-α36 and its effect on SGK1 expression, as well as the phosphorylation of Raf, MEK1/2, and Erk1/2 in SGC7901 cells. β-actin was used as the loading control. (**D**) qRT-PCR analysis of the knockdown efficiency of ER-α36 and the mRNA levels of MAPK1 following ER-α36 knockdown in SGC7901 cells. (**E**) Western blot analysis showing the overexpression efficiency of SGK1 and its effect on the phosphorylation of Raf, MEK1/2, and Erk1/2 in HGC27 cells. β-actin was used as the loading control. (**F**) qRT-PCR analysis of the overexpression efficiency of SGK1 and the mRNA levels of MAPK1 following SGK1 overexpression in HGC27 cells. (**G**) Western blot analysis showing the knockdown efficiency of SGK1 and its effect on the phosphorylation of Raf, MEK1/2, and Erk1/2 in SGC7901 cells. β-actin was used as the loading control. (**H**) qRT-PCR analysis of the knockdown efficiency of SGK1 and the mRNA levels of MAPK1 following SGK1 knockdown in SGC7901 cells. All quantitative data are presented as the mean ± standard deviation (SD) from three independent biological replicates. Statistical significance was evaluated using a two-tailed unpaired Student’s *t*-test. * *p* < 0.05, ** *p* < 0.01, *** *p* < 0.001.

Based on the coordinated upregulation and physical interaction between ER-α36 and SGK1 observed in clinical tissues, we next explored whether ER-α36 modulates the Raf/MEK1/2/Erk1/2 signaling pathway through SGK1 in gastric cancer cells. In HGC27 cells, assays showed that ectopic overexpression of ER-α36 significantly increased SGK1 abundance and enhanced the phosphorylation of Raf, MEK1/2, and Erk1/2, without altering total Erk1/2 protein levels (Figure 3A). To clarify whether this regulation occurs at the transcriptional level, we performed qRT-PCR, which confirmed successful overexpression of ER-α36 but revealed no significant change in MAPK1 mRNA levels (Figure 3B). To further corroborate this regulatory axis, we performed loss-of-function analysis in SGC7901 cells. Knockdown of ER-α36 markedly suppressed SGK1 expression and reduced the phosphorylation levels of Raf, MEK1/2, and Erk1/2, while total Erk1/2 protein remained unchanged (Figure 3C). Correspondingly, qRT-PCR verified efficient silencing of ER-α36 but showed no significant alterations in MAPK1 transcription (Figure 3D; * *p* < 0.05, ** *p* < 0.01, *** *p* < 0.001).

To build on these findings, we next sought to confirm whether SGK1 directly regulates the Raf/MEK1/2/Erk1/2 signaling axis in gastric cancer cells. In HGC27 cells, overexpression of SGK1 (Figure 3E) significantly enhanced the phosphorylation of Raf, MEK1/2, and Erk1/2, whereas qRT-PCR analysis revealed no significant alterations in MAPK1 mRNA levels (Figure 3F). In SGC7901 cells, targeted knockdown of SGK1 (Figure 3G) markedly suppressed the phosphorylation of these kinases, and qRT-PCR further verified that MAPK1 transcription levels remained unchanged (Figure 3H; * *p* < 0.05, ** *p* < 0.01, *** *p* < 0.001). Collectively, these quantitative results demonstrated statistically significant differences in kinase phosphorylation (but not in MAPK mRNA expression), confirming that SGK1 acts downstream of ER-α36 to mediate activation of the Raf/MEK1/2/Erk1/2 pathway at the post-translational level in gastric cancer cells.

To validate the evolutionary conservation of this ER-α36/SGK1/Erk1/2 regulatory axis across species, we conducted complementary experiments in murine MFC gastric cancer cells. Ectopic overexpression of ER-α36 in MFC cells significantly increased SGK1 expression and Erk1/2 phosphorylation, whereas ER-α36 knockdown led to a reduction in both (Figure S2A–D; * *p* < 0.05, ** *p* < 0.01, *** *p* < 0.001). Further mechanistic analysis in MFC cells confirmed that SGK1 itself functions as a core regulator of Erk1/2 phosphorylation: SGK1 overexpression enhanced Erk1/2 phosphorylation, while SGK1 knockdown inhibited it (Figure S3A–D; * *p* < 0.05, ** *p* < 0.01, *** *p* < 0.001). These observations were consistent with our findings in human gastric cancer cell lines, confirming the conserved role of the ER-α36/SGK1/Erk1/2 axis.

These data demonstrated that the ER-α36–SGK1–Erk1/2 regulatory module is evolutionarily conserved across human and mouse gastric cancer cells.

### 3.4. SGK1 Mediate EGF-Induced Erk1/2 Activation in SGC7901 Cells

**Figure 4:**
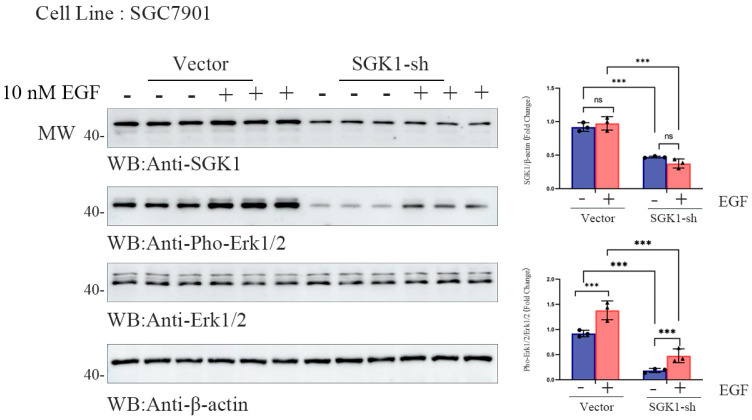
SGK1 mediates stimulation of Erk activation by EGF. Western blot analysis of SGK1 and phosphorylated Erk1/2 (p-Erk1/2) in SGC7901 cells under EGF stimulation. Cells were divided into two groups: empty vector control (Vector) and SGK1 knockdown (SGK1-sh). Cells were treated with or without 10 nM EGF for 10 min before harvest. Total Erk1/2 and β-actin were used as internal controls. Representative blots from three independent experiments are shown. Quantification of SGK1 protein levels and p-Erk1/2 is relative to total Erk1/2 (*n* = 3 biological replicates per group). The data are shown as the mean ± SD with unpaired two-tailed Student’s *t*-test; *** *p* < 0.001.

To explore the role of SGK1 in EGF-induced Erk1/2 activation in gastric cancer, we performed Western blot analysis in SGC7901 cells. We first confirmed that 10 nM EGF stimulation induced Erk1/2 phosphorylation in a time-dependent manner, peaking at 10–20 min, and that this effect was also dose-dependent. We therefore selected the 10 nM EGF and 10 min stimulation conditions for subsequent experiments. Western blot analysis was then performed in SGC7901 cells stably transfected with an empty vector or SGK1 shRNA (Figure S2A). After 10 nM EGF stimulation for 10 min, cell lysates were collected and analyzed to measure SGK1 and phosphorylated Erk1/2 (p-Erk1/2) expression, with total Erk1/2 and β-actin used as internal controls (Figure 4). Quantification of band intensities confirmed that EGF treatment significantly increased p-Erk1/2 levels in control cells, whereas this increase was markedly attenuated in SGK1-knockdown cells (Figure 4; *** *p* < 0.001). These results indicate that SGK1 contributes significantly to EGF-induced Erk1/2 phosphorylation in SGC7901 cells.

These findings were supported by supplementary experiments in MFC cells that confirmed that EGF (10 nM) markedly induced Erk1/2 phosphorylation, and SGK1 knockdown significantly attenuated this effect (Figure S4A,B; * *p* < 0.05, ** *p* < 0.01, *** *p* < 0.001). Thus, SGK1 plays a key role in mediating EGF-induced Erk1/2 activation in gastric cancer cells.

### 3.5. ER-α36 Promotes the Migration, Proliferation and Invasion of SGC7901 Cells Through an SGK1-Dependent Mechanism

**Figure 5:**
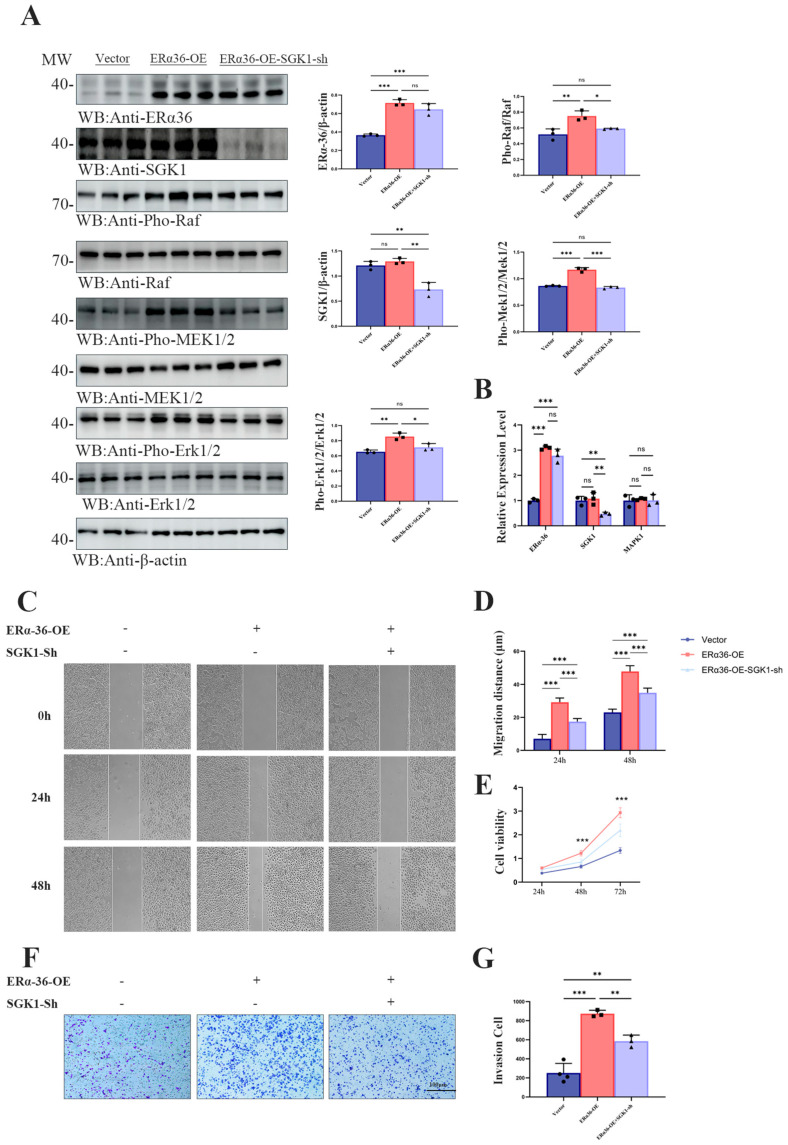
ERα36 promotes the migration, proliferation and invasion of SGC7901 cells through regulating SGK1. (**A**) Western blot analysis showing the protein expression levels of ER-α36, SGK1, and the phosphorylation status of Raf, MEK1/2, and Erk1/2 in SGC7901 cells stably transfected with empty vector (Vector), ER-α36 overexpression plasmid (ERα36-OE), or ERα36 overexpression plus SGK1 knockdown plasmid (ERα36-OE-SGK1-sh). β-actin was used as the loading control. Representative blots from three independent experiments are shown. Quantitative analysis of the relative expression levels of ER-α36, SGK1, p-Raf, p-MEK1/2, and p-Erk1/2, normalized to the corresponding total protein or β-actin level. The three colors correspond to the three experimental groups (Vector, ERα36-OE, and ERα36-OE + SGK1-sh), and their meanings are fully described in the revised legend.Data are presented as the mean ± SD (n = 3 per group). (**B**) The efficiency of ERα36 overexpression and SGK1 knockout, and the qRT-PCR analysis of Mapk1 mRNA level after ER-α36 overexpression (ERα36-OE) or ERα36 overexpression plus SGK1 knockout (ERα36-OE-SGK1-sh) in SGC7901 cells. (**C**) Representative phase-contrast images of the wound-healing (scratch) assay in SGC7901 cells expressing Vector, ERα36-OE, ERα36-OE-SGK1-sh. Images were captured at 0, 24, and 48 h after scratching. (**D**) Quantification of the relative migration distance at 24 and 48 h. The migration rate was calculated as the percentage of wound closure relative to the initial wound width. Data are presented as the mean ± SD (n = 3 biological replicates per group). (**E**) Cell proliferation assessed by the MTT assay. The viability of SGC7901 cells in the three groups was measured at 24, 48, and 72 h. Absorbance at 490 nm is proportional to the number of viable cells. Data are presented as the mean ± SD. (**F**) Representative images of Transwell invasion assays in SGC7901 cells. Invaded cells were fixed, stained with crystal violet, and photographed. Scale bar, 100 μm. (**G**) Quantification of invaded cells. The number of invasive cells was counted in five random fields per chamber. Data are presented as the mean ± SD (n = 3 biological replicates per group). Statistical significance was determined by one-way ANOVA followed by Tukey’s post hoc test. * *p* < 0.05, ** *p* < 0.01, *** *p* < 0.001.

To investigate whether SGK1 is required for ER-α36 to drive gastric cancer cell migration, proliferation, and invasion, we performed gain- and loss-of-function assays in SGC7901 cells. SGC7901 cells were divided into three groups: empty vector control (Vector), stable ER-α36 overexpression (ERα36-OE), and ERα36-OE plus SGK1 knockdown (ERα36-OE + SGK1-sh). The activation of the Raf/MEK/Erk1/2 pathway, cell migration, proliferation, and invasion were evaluated by Western blot, wound healing, MTT, and Transwell assays, respectively. First, Western blot analysis showed that ER-α36 overexpression significantly increased the phosphorylation of Raf, MEK1/2, and Erk1/2, without affecting the total levels of these kinases. SGK1 knockdown in ERα36-OE cells partially attenuated these increases, confirming that ER-α36 activates the Raf/MEK/Erk1/2 pathway, at least in part, through SGK1 (Figure 5A). In order to clarify whether this regulation occurs at the transcription level, we carried out qRT-PCR, which confirmed that ERα36 was successfully overexpressed and SGK1 was knocked down, but there was no significant change in MAPK1 mRNA level (Figure 5B).

The wound-healing assay revealed that ER-α36 overexpression significantly accelerated cell migration at both 24 h and 48 h, as shown by a larger reduction in the wound area compared with the control (Figure 5C,D). SGK1 knockdown markedly reduced this enhanced migratory phenotype, indicating that SGK1 contributes to ER-α36-mediated cell migration. Cell proliferation was assessed using the MTT assay over 72 h. ER-α36-OE cells exhibited significantly higher viability than control cells at 48 h and 72 h, while SGK1 knockdown in ERα36-OE cells significantly attenuated this proliferation advantage (Figure 5E). Consistent with these findings, Transwell invasion assays showed that ER-α36 overexpression significantly increased the number of invaded cells, whereas concomitant SGK1 knockdown reversed this effect (Figure 5F,G).

Taken together, these results suggest that SGK1 plays a significant role in ER-α36-mediated migration, proliferation, and invasion of SGC7901 cells.

### 3.6. ERα-36 Drives Gastric Tumor Growth and EGFR/Raf/MEK/ERK Activation In Vivo, Which Is Attenuated by SGK1 Knockdown

**Figure 6:**
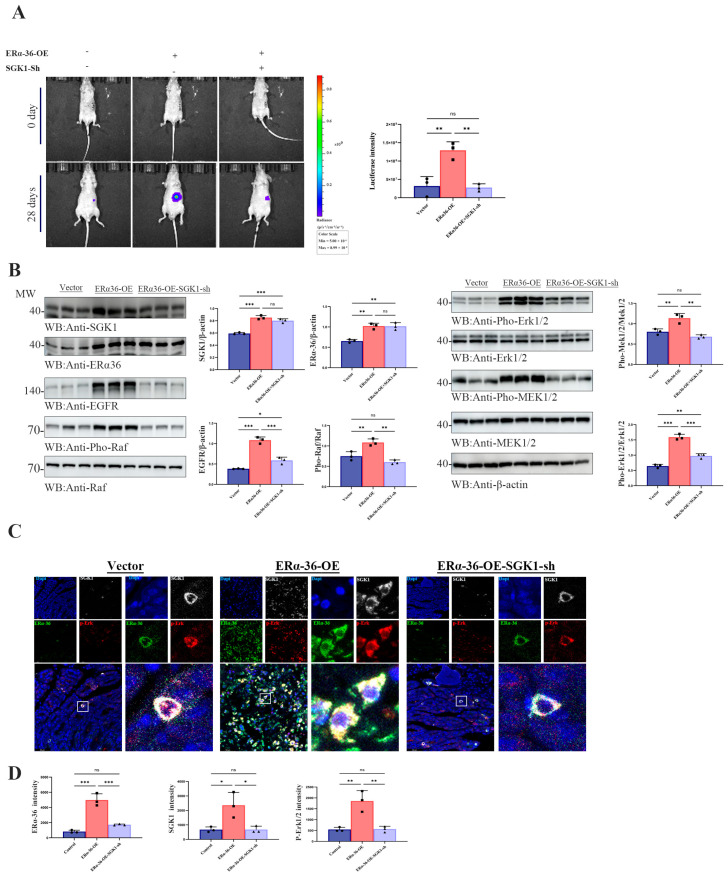
ER-α36 promoted tumor growth and activated EGFR/Raf/MEK/ERK signaling pathway in vivo, which was weakened by SGK1 knockout. (**A**) Bioluminescence imaging (BLI) monitoring of orthotopic gastric tumor growth in nude mice. SGC7901 cells stably expressing luciferase, overexpressing ERα36 (ERα36-OE) and overexpressing ERα36 with SGK1 (ERα36-OE-SGK1-KO) were orthotopically implanted into the stomach wall of BALB/c nude mice (n = 3 per group). Tumor progression was tracked by BLI at day 0 and day 28 post-inoculation. Representative bioluminescence images for each group are shown. (**B**) Western blot analysis of key signaling molecules in tumor tissues. Tumor lysates were extracted from the xenograft models. Protein expression of SGK1, ER-α36, EGFR, as well as components of the Raf/MEK/ERK pathway (Raf, MEK1/2, Erk1/2) was detected by Western blotting. β-actin was used as the loading control. (**C**) Immunofluorescence (IF) staining of ER-α36, SGK1, and p-Erk1/2 in tumor tissues. Representative IF images showing the subcellular localization and co-expression of ER-α36 (green), SGK1 (gray), and p-Erk1/2 (red) in tumor tissues harvested from the indicated groups. Nuclei were counterstained with DAPI (blue). The white squares mark the magnified regions of interest (ROIs) shown in the corresponding panels. (**D**) Quantification of IF fluorescence intensity in tumor tissues. The fluorescence signals of ER-α36, SGK1, and p-Erk1/2 were quantified using ImageJ software. Data are presented as the mean ± standard deviation (SD) from three independent biological replicates. Statistical significance was evaluated using an unpaired two-tailed Student’s *t*-test. * *p* < 0.05, ** *p* < 0.01, *** *p* < 0.001.

To validate the functional relevance of the ER-α36/SGK1/EGFR/Raf/MEK/ERK axis in vivo, we next investigated whether ER-α36 promotes gastric tumor growth through SGK1-mediated activation of this signaling pathway, and whether SGK1 knockdown could attenuate these pro-tumorigenic effects. Bioluminescence imaging was used to dynamically monitor tumor growth in an orthotopic gastric cancer xenograft model: SGC7901 cells with stable luciferase expression, combined with ER-α36 overexpression and/or SGK1 knockdown, were orthotopically injected into the gastric wall of nude mice (n = 3 per group). Tumor growth was non-invasively tracked by bioluminescence imaging at baseline (day 0) and 28 days post-inoculation, with representative imaging results for each group presented (Figure 6A). To further elucidate the underlying molecular mechanisms in vivo, we performed Western blot analysis on tumor lysates from orthotopic xenografts to assess the expression of ER-α36, SGK1, EGFR, and key phosphorylated components of the Raf/MEK/ERK signaling pathway across experimental groups. Consistent with the in vitro findings, ER-α36 overexpression significantly upregulated SGK1 expression and enhanced the phosphorylation (activation) of the EGFR/Raf/MEK/ERK signaling cascade in xenograft tissues. Conversely, SGK1 knockdown markedly reduced the activation of this pathway and abrogated the regulatory effect of ER-α36 on EGFR signaling (Figure 6B).

To further characterize the spatial expression patterns and functional coordination of ER-α36, SGK1, and p-Erk1/2 in vivo, we analyzed their protein expression and subcellular localization in nude mouse orthotopic xenograft tissues. ER-α36 overexpression significantly upregulated the expression of SGK1 and p-Erk1/2 in xenograft tumors, and this pro-activation effect was abrogated by SGK1 knockdown. Representative immunofluorescence staining images visualizing the subcellular localization and co-expression profiles of ER-α36 (green), SGK1 (gray), and p-Erk1/2 (red) in xenograft tissues from the control, ERα36-OE, and ERα36-OE plus SGK1-Sh groups, with nuclei counterstained by DAPI (blue), are shown in Figure 6C. To quantify these expression changes, the protein levels of ER-α36, SGK1, and p-Erk1/2 in xenograft tissues were normalized to adjacent non-tumorous control tissues, and statistical analysis was performed using an unpaired two-tailed *t*-test (Figure 6D; * *p* < 0.05, ** *p* < 0.01, *** *p* < 0.001). The quantitative results confirmed that ER-α36 overexpression markedly enhanced the co-expression of ER-α36, SGK1, and p-Erk1/2, with coordinated subcellular localization in xenograft tissues, whereas concurrent SGK1 knockdown significantly suppressed this co-expression trend.

## 4. Discussion

ER-α36 represents a unique membrane-associated estrogen receptor that mediates rapid non-genomic signaling independent of classical nuclear transcriptional activity. Mounting evidence has demonstrated that ER-α36 is abnormally upregulated in multiple human malignancies [20,21], and promotes cancer cell proliferation, migration, and therapeutic resistance through crosstalk with membrane receptor tyrosine kinases [22,23]. However, the vast majority of studies focused on breast cancer and glioblastoma, in which, ER-α36 directly interacts with EGFR to trigger downstream MAPK/Erk signaling [2,5]. Whether such a regulatory mode exists in gastric cancer, and whether a specific kinase mediates this signaling process, remains largely unexplored. The present study extends our understanding of ER-α36 signaling by identifying SGK1 as a key downstream mediator that links ER-α36 to EGFR-Erk1/2 activation in gastric cancer.

In the present study, immunohistochemical analysis demonstrated that ER-α36 was positively expressed in 62.3% of gastric adenocarcinoma tissues and was significantly associated with poor histological differentiation, but not with other clinicopathological parameters. These clinical observations suggest that ER-α36 may participate in the loss of differentiation during gastric cancer progression, which is consistent with its role as a driver of malignant phenotypes in other tumor types [24]. Kaplan–Meier survival analysis further indicated that high ER-α36 expression predicted unfavorable prognosis, supporting its potential value as a prognostic biomarker for gastric adenocarcinoma.

Bioinformatics analysis using the GSA database (accession No. HRA001689) systematically screened kinases associated with ER-α36 and identified SGK1 as the most prominent candidate. SGK1 is a serine/threonine kinase structurally related to AKT, and its dysregulation has been widely reported in various cancers [9,17], and studies have linked high SGK1 expression to poor tumor prognosis in gastric cancer [25]. SGK1 promotes cell proliferation and migration by enhancing EGFR signaling activity [26,27]. However, no studies have established a connection between ER-α36 and SGK1. Our immunofluorescence assays showed that ER-α36 and p-Erk1/2 exhibited obvious co-localization in gastric cancer tissues while ER-α36, SGK1, and p-Erk1/2 were coordinately upregulated in tumor cells. More importantly, co-immunoprecipitation assays verified a direct physical interaction between ER-α36 and SGK1, which provides a molecular basis for their functional cooperation.

At the cellular level, we systematically verified the regulatory hierarchy of the ER-α36–SGK1–Erk1/2 axis. Overexpression or knockdown of ER-α36 significantly altered SGK1 expression and the phosphorylation status of Raf, MEK1/2, and Erk1/2. Conversely, manipulation of SGK1 expression directly affected Erk1/2 phosphorylation without being influenced by ER-α36 in a feedback manner. Notably, SGK1 regulated Erk1/2 activity at the post-translational level rather than through transcriptional control, confirming its role as a signaling kinase. This regulatory pattern was also observed in human gastric cancer cell lines (SGC7901, HGC27, and NCI-N87) and mouse MFC cells, indicating evolutionary conservation of this signaling axis.

EGF-induced EGFR activation plays a critical role in gastric cancer progression [4]. Our results showed that EGF stimulation strongly induced Erk1/2 phosphorylation, and this effect was significantly inhibited by SGK1 knockdown, indicating that EGF-induced MAPK activation is SGK1-dependent. This finding is distinct from previous reports in other cancer types in which ER-α36 directly binds EGFR to activate Erk signaling [2,5]. Therefore, our study revealed a tumor-type-specific mechanism: in gastric cancer, ER-α36 forms a functional complex with SGK1, which serves as a molecular bridge to transmit signals to the Erk pathway. This discovery expands the mechanistic diversity of ER-α36 signaling and identifies a newly identified ER-α36/SGK1/Erk regulatory axis in gastric cancer.

Functional experiments demonstrated that ER-α36 overexpression promoted gastric cancer cell proliferation, migration, and invasion, whereas SGK1 knockdown completely abolished these oncogenic effects. In vivo orthotopic xenograft models further confirmed that ER-α36 promoted gastric tumor growth and enhanced the phosphorylation of EGFR, Raf, MEK1/2, and Erk1/2, which were reversed by SGK1 silencing. These gain- and loss-of-function data solidly establish SGK1 as a critical downstream effector of ER-α36-driven malignant phenotypes.

The Erk signaling pathway is a core intracellular cascade that regulates multiple biological processes including cell proliferation, differentiation, and survival [28,29]. In gastric cancer, aberrant Erk activation is closely associated with tumor invasion, metastasis, and poor prognosis [30]. Our study identified a novel upstream regulatory mechanism of the Erk pathway mediated by the ER-α36–SGK1 axis, which provides a new molecular explanation for abnormal Erk activation in gastric cancer.

Several limitations of this study should be acknowledged. First, the clinical sample size (n = 53) was relatively small, which limited the statistical power for correlation analysis between SGK1 and comprehensive clinicopathological features. Second, the precise binding domains and post-translational modifications between ER-α36 and SGK1 remain to be elucidated. Third, pharmacological inhibition of SGK1 and combination therapy with EGFR inhibitors were not performed. Future studies will employ large-scale clinical cohorts, structural biology analyses, and selective SGK1 inhibitors (GSK650394) to further validate and translate this signaling axis.

This study identified and validated a novel gastric cancer-specific signaling axis: ER-α36–SGK1–Erk1/2. ER-α36 physically interacts with SGK1 and mediates EGF-EGFR-induced Erk1/2 activation in an SGK1-dependent manner, thereby promoting gastric cancer proliferation, invasion, and tumor growth. These findings deepen our understanding of non-genomic estrogen signaling in gastric malignancies and provide a promising therapeutic target for gastric cancer intervention.

## 5. Conclusions

In summary, the present study identified ER-α36 as a critical oncogenic driver in gastric adenocarcinoma that functions by upregulating SGK1 to sustain activation of the Raf/MEK/Erk1/2 signaling axis. We demonstrated that ER-α36, SGK1, and p-Erk1/2 are coordinately overexpressed in gastric cancer and correlate with poor histological differentiation and unfavorable patient survival. Mechanistically, ER-α36 physically interacts with SGK1 and enhances EGFR-dependent Erk1/2 phosphorylation in an SGK1-facilitated manner. Functionally, ER-α36 promotes gastric cancer cell proliferation, migration, invasion, and orthotopic tumor growth, whereas SGK1 knockdown markedly attenuates these malignant phenotypes. Collectively, our findings establish the ER-α36–SGK1–Erk1/2 axis as a novel and functionally critical signaling cascade in gastric cancer progression. Targeting this pathway may represent a promising therapeutic strategy for gastric cancer.

## Figures and Tables

**Table 1:** Relationship between expressed level of ER-α36 protein and clinicopathological characteristics.

Factor	ER-α36		
	Positive	Negative	*p*-Value
Age			0.70
≤60	28	17	
>60	5	3	
Gender			0.52
Male	11	5	
Female	22	15	
Tumor size			0.47
≤5 cm	23	12	
>5 cm	10	8	
Histological differentiation			0.02 *
High differentiation	12	14	
Low differentiation	21	6	
T stage			0.70
T2 T3	10 8	4 6	
T4	15	10	
N stage			0.75
N0	7	5	
N1–3	26	15	

Note: Histological differentiation was evaluated based on the 2003 WHO clinicopathological criteria for gastric adenocarcinoma. High differentiation (well-differentiated) means that tumor cells are highly similar to normal gastric glandular epithelial cells with an intact glandular structure, regular arrangement and mild nuclear atypia. Low differentiation (poorly differentiated) refers to significant differences between tumor cells and normal gastric epithelial cells, with disordered or absent glandular structure, obvious nuclear atypia and frequent mitotic figures. * Indicates a statistically significant difference between groups.

## Data Availability

The original contributions presented in this study are included in the article/Supplementary Materials. Further inquiries can be directed to the corresponding authors.

## Supplementary Materials

The following supporting information can be downloaded at: https://www.mdpi.com/article/10.3390/cells15090787/s1.
