# Supplementary material for: Estrogen Receptor-α36 Mediates EGFR-SGK1 Signaling-Related Erk Activation in Gastric Cancer
Source: Cells. 2026 Apr 26;15(9):787. doi: 10.3390/cells15090787 (PMC13162801; doi:10.3390/cells15090787)
Supplement: Supplementary file 1 [file cells-15-00787-s001.zip › cells-4238380-supplementary.pdf]

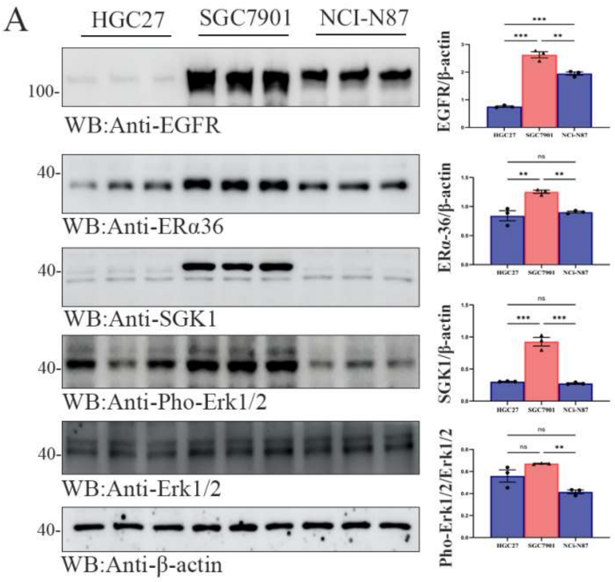

Figure S1: Western blot analysis of protein expression levels in gastric cancer cell lines. **(A)** EGFR, ER-α36, and SGK1 protein expression was detected in three human gastric cancer cell lines (HGC27, SGC7901, and NCI-N87). (n=3–6 biological replicates; mean ± SD; unpaired two-tailed t-test, *p<0.05, **p<0.01, ***p<0.001)

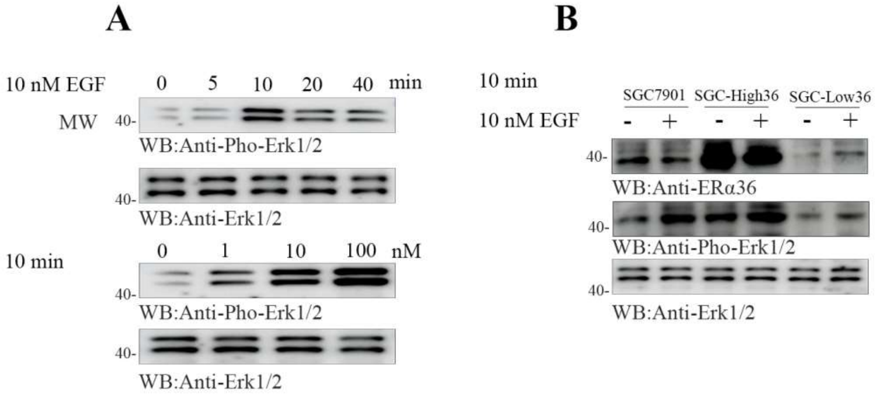

Figure S2: ER-α36 modulates EGF-induced Erk1/2 phosphorylation in SGC7901 gastric cancer cells. **(A)** Time- and dose-dependent induction of Erk1/2 phosphorylation by EGF in SGC7901 cells.Cells were treated with 10 nM EGF for the indicated durations (0–40 min). Erk1/2 phosphorylation peaked at 10–20 min. Cells were treated with the indicated concentrations of EGF (0–100 nM) for 10 min. Erk1/2 phosphorylation increased in a dose-dependent manner.Total Erk1/2 served as the loading control for both panels. Representative blots from three independent experiments are shown.**(B)** Effect of ER-α36 expression level on EGF-induced Erk1/2 phosphorylation.SGC7901 cells (control), SGC7901 cells with stable ER-α36 overexpression (SGC-High36), and SGC7901 cells with stable ER-α36 knockdown (SGC-Low36) were treated with or without 10 nM EGF for 10 min.Blots were probed for ER-α36, phosphorylated Erk1/2 (p-Erk1/2), and total Erk1/2. Total Erk1/2 served as the loading control. Representative blots from three independent experiments are shown.

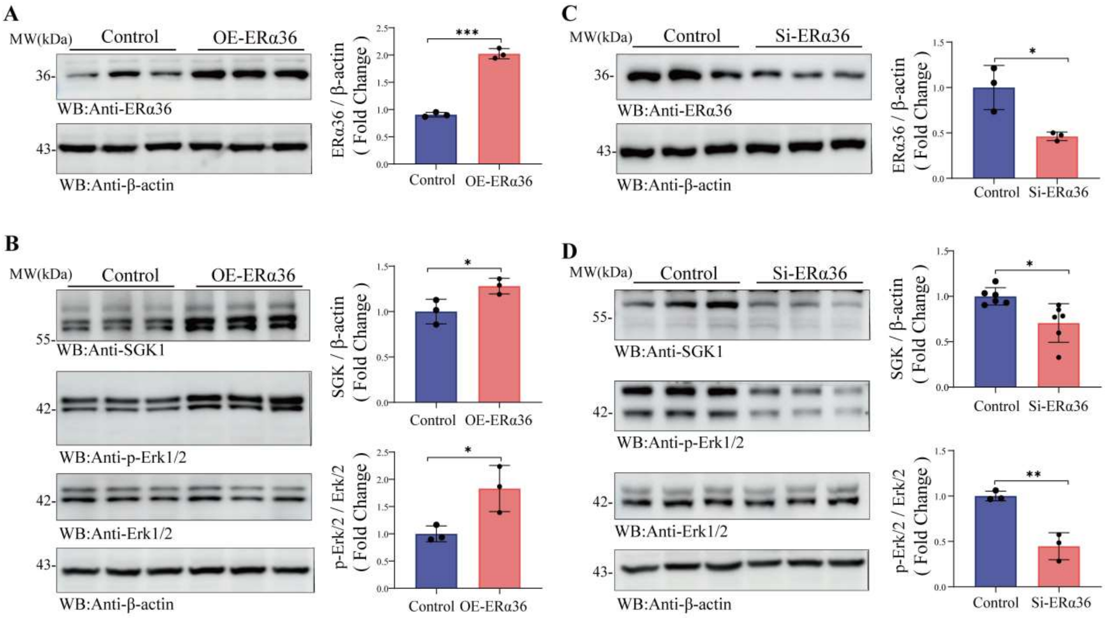

Figure S3: ER-α36 promoted the phosphorylation of Erk1/2 via SGK1 in MFC cells. (A) The over-expression efficiency of ER-a36 as well as (**B)** the influence of ER-a36 over-expression on SGK1 and the phosphorylation of Erk1/2 in MFC cell line were detected by western blot (n=3 biologically independent samples per group). **(C)** The down-expression efficiency of ER-a36 as well as (**D)** Western blot analysis of SGK1 and p-ERK1/2 in MFC cells following ER-α36 knockdown (n=3–6 biological replicates; mean ± SD; unpaired two-tailed t-test, *p<0.05, **p<0.01, ***p<0.001).

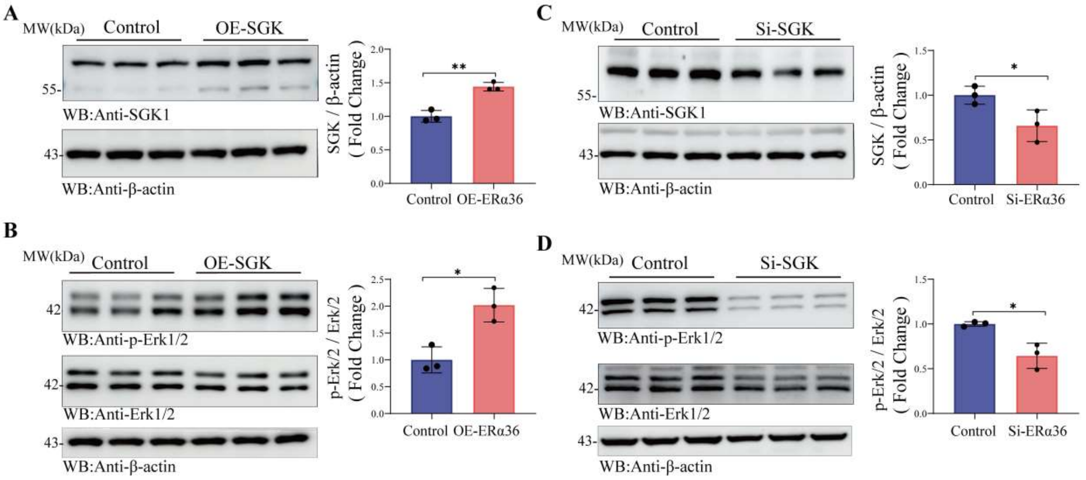

Figure S4: SGK1 promoted the phosphorylation of Erk1/2 in MFC cells. (A) The over-expression efficiency of SGK as well as (**B)** Western blot analysis of SGK1 and p-ERK1/2 in MFC cells following ER-α36 knockdown (n=3–6 biological replicates; mean ± SD; unpaired two-tailed t-test, *p<0.05, **p<0.01, ***p<0.001). **(C)** The down-expression efficiency of SGK as well as (**D)** Western blot analysis revealed that SGK1 knockdown modulated p-ERK1/2 levels in MFC cells (n=3 biological replicates; mean±SD). Statistical significance was assessed by two-tailed unpaired t-test(*p<0.05,**p<0.01,***p<0.001).

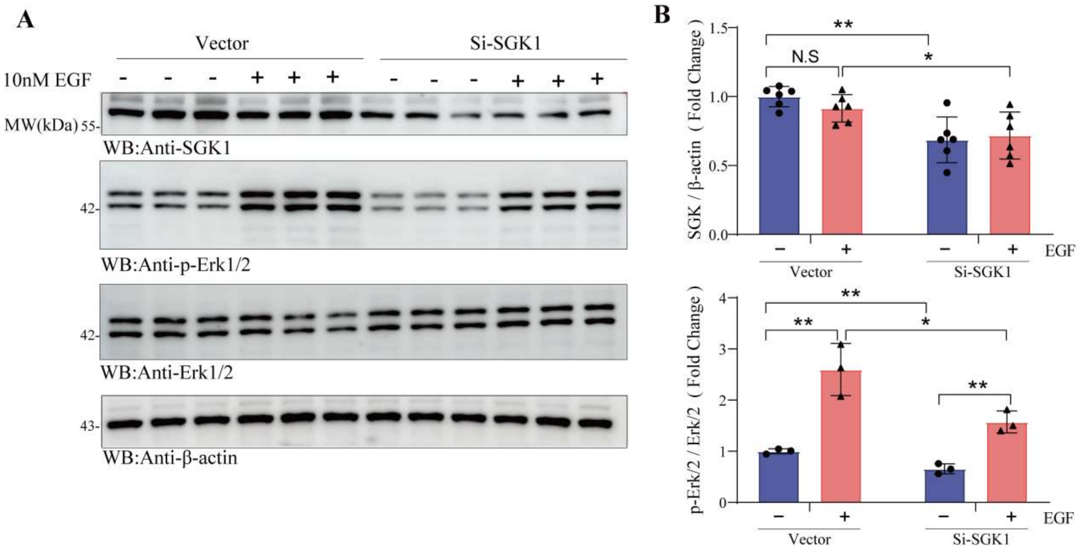

Figure S5: EGF promoted the phosphorylation of Erk1/2 independent on SGK in MFC cells. (A) The influence of EGF (100 ng/mL) as well as down-expression on SGK1 on the phosphorylation of Erk1/2 in MFC cell line were detected by western blot. **(B)** SGK1 protein levels and p-ERK1/2 were analyzed by Western blot (n=3-6 biological replicates per group).The data are shown as mean ± SD with unpaired two-tailed Student's t-test,*p<0.05, **p<0.01, ***p<0.001).
